# LSL-YOLO11n: a YOLO11n-based model for maize leaf disease detection in complex field environments

**DOI:** 10.3389/fpls.2026.1840425

**Published:** 2026-06-16

**Authors:** Chengwen Yang, Qisheng Feng, Jingjing Mai, Ruoqi Zhang, Ni Chong, Zhicheng Ru, Tiangang Liang

**Affiliations:** 1 State Key Laboratory of Herbage Improvement and Grassland Agro-ecosystems, Key Laboratory of Grassland Livestock Industry Innovation, Ministry of Agriculture and Rural Affairs, College of Pastoral Agriculture Science and Technology, Lanzhou University, Lanzhou, China; 2 Center for Remote Sensing of Ecological Environments in Cold and Arid Regions, Lanzhou University, Lanzhou, China

**Keywords:** complex field environments, localization quality estimation, LSL-YOLO11n, maize leaf disease detection, object detection, YOLO11n

## Abstract

Maize leaf diseases in field environments often exhibit large variations in lesion scale, irregular morphology, blurred boundaries, and complex backgrounds. These factors pose challenges for existing detection models, particularly in detecting small lesions and achieving precise bounding-box localization. To address these issues, this study proposes LSL-YOLO11n, a maize leaf disease detection model based on the YOLO11n framework. The proposed model improves feature representation, localization quality modeling, and bounding-box regression to enhance disease detection performance under complex field conditions. Experiments were conducted on a dataset containing 15,119 images and 29,366 annotated instances across eight categories, including seven maize disease categories and healthy leaves. To evaluate the effectiveness of the proposed model, ablation experiments, comparative experiments with mainstream object detection models, and visual detection analyses were carried out. The ablation results show that the improved components contribute positively to the overall detection performance. LSL-YOLO11n achieves a Precision of 84.4%, Recall of 73.9%, and mean Average Precision (mAP) of 83.3%, which is 3.1 percentage points higher than that of the baseline YOLO11n model. Compared with YOLOv8n, YOLOv9t, YOLOv10n, and YOLOv12n, the proposed model improves mAP by 4.7, 3.3, 5.3, and 10.9 percentage points, respectively. The visual detection results further indicate that LSL-YOLO11n performs more stably in complex backgrounds and small-lesion scenarios. These findings provide technical support for rapid maize disease recognition and intelligent field monitoring.

## 1 Introduction

Maize is one of the major food crops in China and plays a crucial role in ensuring national food security. Due to its wide cultivation and high yield, maize is of great importance in food production, animal feed, and industrial raw materials. In recent years, influenced by factors such as climate change, farming practices, and pathogen transmission, the frequency and severity of maize diseases have shown an increasing trend (Wang et al., 2022a). Among them, leaf diseases such as gray leaf spot, northern corn leaf blight, and rust occur frequently and cause severe damage, posing significant threats to plant photosynthesis, physiological functions, and ultimately crop yield (Li et al., 2025a).

In real field environments, disease lesions are often small in scale, irregular in shape, and blurred in boundary, and are frequently affected by illumination variation, occlusion, and complex backgrounds (Barbedo, 2019; Timilsina et al., 2025). These factors make accurate localization and stable identification of maize diseases more challenging (Timilsina et al., 2025). Meanwhile, due to limitations in computational resources and deployment conditions of field equipment, efficient deep learning models have attracted increasing attention. Owing to their potential advantages in inference efficiency, such models have become an important research direction for disease detection in intelligent agriculture, especially for scenarios where computational resources may be limited (Li et al., 2025b). Therefore, developing disease detection methods with high accuracy and strong robustness under complex field environments, enabling precise localization and reliable recognition of multi-scale lesions, has become an important technological demand in smart agricultural disease monitoring and management (Zhao et al., 2025).

Early maize disease recognition mainly relied on manual inspection. This approach is not only time-consuming and labor-intensive, but its effectiveness also depends heavily on the experience of the operator, resulting in strong subjectivity and instability. Consequently, consistent recognition accuracy cannot be guaranteed, and delayed manual identification may lead to missed opportunities for optimal disease control (Shoaib et al., 2023). In recent years, with the development of image processing and computer technologies, image recognition techniques have provided theoretical and technical support for the automatic identification of maize diseases (Nyawose et al., 2025). Deep learning-based image recognition methods can help farmers rapidly and accurately identify maize diseases, enabling timely intervention and thus effectively improving maize yield and quality (Mohanty et al., 2016; Ferentinos, 2018; Khan et al., 2023).

With the rapid development of smart agriculture, the use of computer vision technologies for automatic crop disease diagnosis has become a major research focus worldwide. Early studies mainly relied on traditional machine learning algorithms such as Support Vector Machines (SVM) and Random Forests. These methods depend heavily on manually designed features, such as color histograms and texture descriptors. However, their generalization ability in complex field environments is relatively poor, and they often fail to meet the requirements of real-time detection (Dang et al., 2024). In recent years, deep learning technologies represented by Convolutional Neural Networks (CNNs) have achieved significant breakthroughs in robust feature extraction and have gradually become the mainstream methods in this field (Ferentinos, 2018; Too et al., 2019; Li et al., 2022; Theerthagiri et al., 2024). Deep learning-based object detection methods provide an effective framework for localizing and recognizing disease targets in complex images, and have been widely developed from two-stage detectors to efficient one-stage detection frameworks (Ren et al., 2017; Zhao et al., 2019; Liu et al., 2020).

Given the limited computational resources available in many agricultural applications, designing models that maintain high accuracy and robustness under constrained computational budgets has become an important research trend (Mittal, 2024). The primary strategy is to enhance the model’s ability to recognize disease lesions in complex backgrounds by optimizing convolution operations and feature fusion structures. For example, mechanisms such as shared convolution and scale-aware feature learning can improve multi-scale feature representation, thereby enhancing lesion detection accuracy (Liu et al., 2025).

Song et al. (2023) addressed the challenges of lesion localization and recognition caused by leaf occlusion and overlap in complex field environments and proposed a cassava leaf disease detection method based on YOLOX. In addition to improving the network architecture, their study introduced Quality Focal Loss (QFL) at the training objective level as an optimization strategy for the classification branch. This approach enables classification scores to reflect not only category discrimination but also the localization quality of detected objects, thereby alleviating the inconsistency between high classification confidence and inaccurate localization. Consequently, the stability and accuracy of disease detection in complex backgrounds were significantly improved. This idea is consistent with the motivation of the present study, which aims to improve classification–localization consistency through localization quality modeling, and thus provides a valuable reference for enhancing detection reliability in agricultural disease detection scenarios.

To meet the real-time detection requirements of maize diseases in field environments, Li et al. (2024) developed a lightweight detection model based on YOLOv8 and introduced efficiency-oriented architectural improvements to enhance mobile-device inference speed and detection robustness. In addition, Ma et al. (2024) addressed challenges such as weak lesion features and unstable localization boundaries in large-scale field environments by incorporating several strategies into YOLOv8, including the Global Attention Mechanism (GAM), Content-Aware ReAssembly of FEatures (CARAFE) upsampling, and Wise-IoU bounding box regression loss. These improvements enhanced the model’s ability to focus on key disease features and improved the quality of bounding box regression, providing useful insights for improving detection robustness and localization accuracy in complex agricultural scenarios.

The performance of object detection models depends not only on network architecture but also on the design of loss functions, especially bounding-box regression losses (Rezatofighi et al., 2019; Zheng et al., 2020; Gevorgyan, 2022; Su et al., 2024). Traditional IoU-based losses, such as Intersection over Union (IoU) and its variants (GIoU and CIoU), mainly measure the overlap area and center distance between predicted and ground-truth bounding boxes. However, during the early stages of training, predicted boxes often exhibit a “wandering” behavior, resulting in slow convergence and reduced regression accuracy (Zheng et al., 2020; Gevorgyan, 2022; Su et al., 2024). The Inner-SIoU loss incorporates both geometric relationships and directional information into bounding box regression while emphasizing the core overlapping regions. Previous studies on IoU-based and SIoU-based regression losses have shown that incorporating geometric constraints, directional information, and region-aware overlap modeling can improve localization stability and detection accuracy (Zheng et al., 2020; Gevorgyan, 2022; Su et al., 2024). Nevertheless, although Inner-SIoU shows promising potential in object detection applications, its performance in dense maize disease detection scenarios still warrants further investigation.

Furthermore, many existing detection models suffer from the misalignment between classification and regression, where bounding boxes with high classification scores are not necessarily the most accurately localized (Fang et al., 2024). Li et al. (2021) proposed improving bounding box regression by introducing localization quality estimation and distribution modeling, which significantly enhances detection performance. This work provides an important reference for integrating localization quality estimation modules into detection frameworks.

In summary, although existing models have partially addressed issues related to accuracy and robustness in maize disease detection, there remains room for improvement in lesion feature extraction under complex backgrounds, precise bounding box regression, and localization quality evaluation (Wang et al., 2024). Therefore, based on the YOLO11n framework, this study proposes an improved detection algorithm that integrates LSCD feature enhancement, LQE localization quality estimation, and the Inner-SIoU loss function, aiming to improve detection accuracy and stability under complex field conditions. Following this research idea, this study first constructs a maize leaf disease detection dataset covering multiple disease categories and complex visual conditions. Then, LSL-YOLO11n is constructed based on the YOLO11n framework by integrating existing improvement modules for feature representation, localization quality modeling, and bounding-box regression. Finally, ablation experiments, comparative experiments with mainstream object detection models, and visual detection analyses are conducted to evaluate the effectiveness and robustness of the proposed model under complex field environments.

## 2 Materials and methods

### 2.1 Data source of maize diseases

This study focuses on maize leaf disease detection. The dataset used in this study contains eight categories: northern corn leaf blight, brown spot, common rust, common smut, downy mildew, grey leaf spot, maize streak disease, and healthy maize leaves. These categories were selected because they represent common maize leaf diseases and can support the evaluation of detection performance under multi-class disease scenarios.

The original images were collected and organized from publicly available maize disease image datasets, mainly including PlantVillage, PlantDoc, and maize disease datasets released on the Kaggle platform. PlantVillage provides widely used maize leaf disease images, including northern corn leaf blight, common rust, grey leaf spot, and healthy maize leaves. PlantDoc contains plant disease images captured under more natural and complex backgrounds, which helps improve the diversity of disease appearance and imaging conditions. In addition, Kaggle maize disease datasets were used to supplement additional disease categories and samples, such as brown spot, common smut, downy mildew, and maize streak disease. After category screening, dataset organization, and annotation verification, the final dataset contained 15,119 images and 29,366 annotated instances across eight categories.

### 2.2 Dataset construction

The dataset was organized in YOLO format for object detection. Each image corresponded to a TXT annotation file, and each line in the annotation file represented one annotated object. The annotation format was defined as class_id, x_center, y_center, width, and height, where the bounding-box coordinates were normalized to the range of 0 to 1. The category indices ranged from 0 to 7, corresponding to the eight maize disease and healthy leaf categories listed in **Table 1**.

**Table 1:** Distribution of annotated instances for maize disease categories in the dataset.

Disease category	Training instances	Validation instances	Test instances	Total
Northern Corn Leaf Blight	2724	808	428	3960
Brown Spot	2224	654	347	3225
Common Rust	3510	1056	503	5069
Common Smut	2853	802	395	4050
Downy Mildew	2470	731	330	3531
Grey Leaf Spot	2727	767	430	3924
Maize Streak Disease	558	152	76	786
Healthy Maize Leaves	3320	1063	438	4821
Total	20386	6033	2947	29366

Instances refer to annotated bounding boxes rather than image numbers. The dataset contains 15,119 images and 29,366 annotated instances in total.

To improve the diversity of training samples and enhance model generalization, data augmentation was applied during model training. Specifically, the default YOLO online augmentation strategy was adopted, including HSV color-space augmentation, random translation, random scaling, horizontal flipping, and Mosaic augmentation. These augmentation operations increased the diversity of image appearances without introducing additional disease categories and helped improve the robustness of the model under variations in illumination, scale, and lesion position.

The dataset was divided into training, validation, and test sets at the image level with a ratio of approximately 7:2:1. Specifically, the training set contained 10,609 images and 20,386 annotated instances, the validation set contained 3,004 images and 6,033 annotated instances, and the test set contained 1,506 images and 2,947 annotated instances. The same data split was used for all comparative and ablation experiments to ensure fairness.

**Figure 1:**
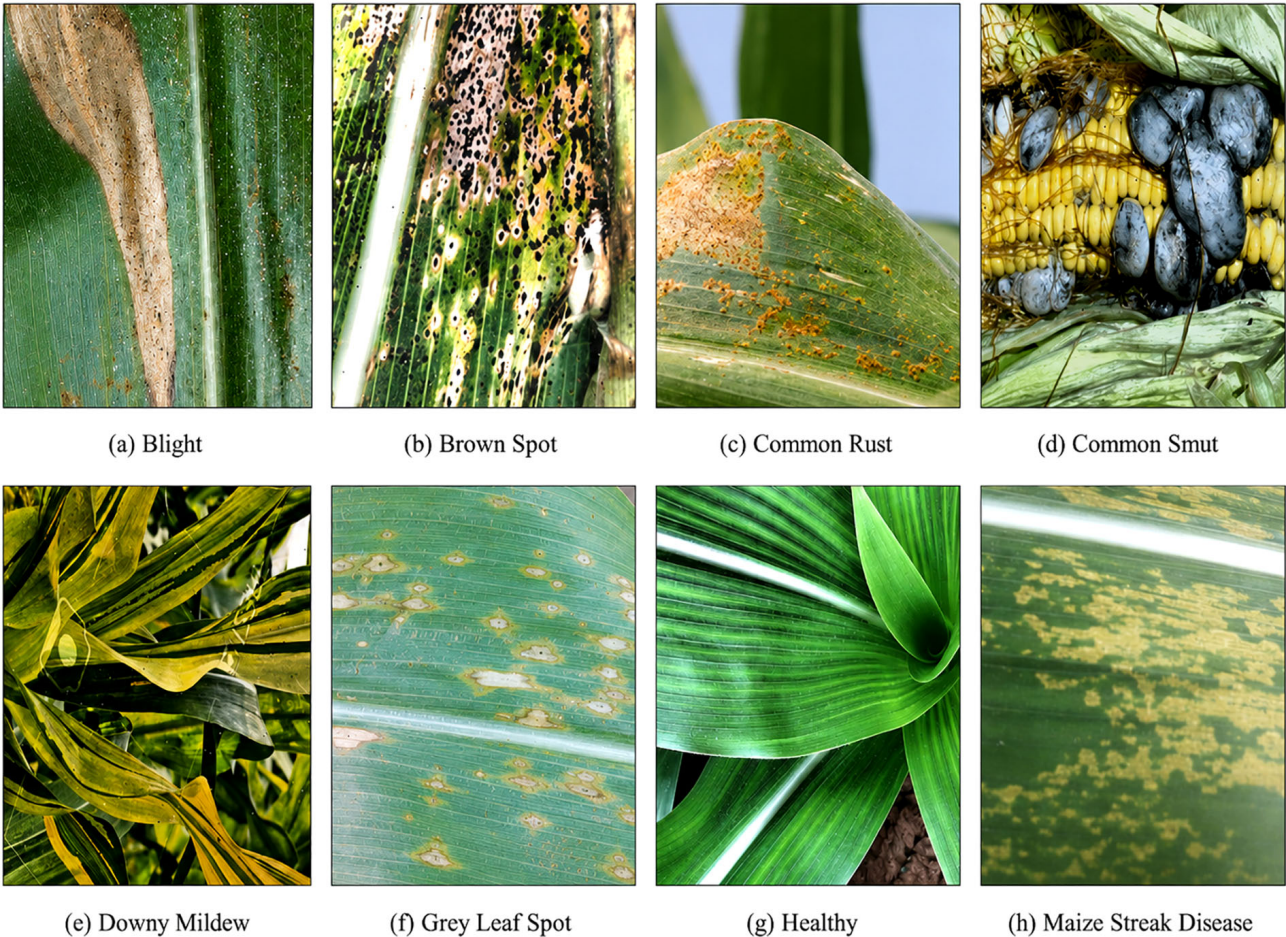
Representative examples of the eight maize leaf categories in the dataset: **(a)** Blight, **(b)** Brown Spot, **(c)** Common Rust, **(d)** Common Smut, **(e)** Downy Mildew, **(f)** Grey Leaf Spot, **(g)** Healthy, and **(h)** Maize Streak Disease.

As shown in **Table 1**, the distribution of annotated instances indicates a certain degree of class imbalance. In particular, maize streak disease had fewer annotated instances than several other categories. To maintain the completeness of maize disease categories, this category was retained in the training, validation, and test sets. Representative examples of the eight maize leaf categories are shown in **Figure 1**.

### 2.3 Introduction to the YOLO11n model

In this study, YOLO11n implemented in the Ultralytics framework was adopted as the baseline detector because it provides a favorable trade-off between detection accuracy and computational efficiency (Ali and Zhang, 2024). The YOLO series has evolved from earlier versions, such as YOLOv3 (Nepal and Eslamiat, 2022), YOLOv4 (Aldakheel et al., 2024), and YOLOv7 (Deng et al., 2024), and has become a widely used framework for real-time object detection. Given its compact model size, efficient inference, and suitability for resource-constrained agricultural applications, YOLO11n was selected as the foundational detection framework. Targeted modifications were then made to the detection head, localization quality estimation, and bounding-box regression strategy to improve detection accuracy and robustness in complex field environments.

**Figure 2:**
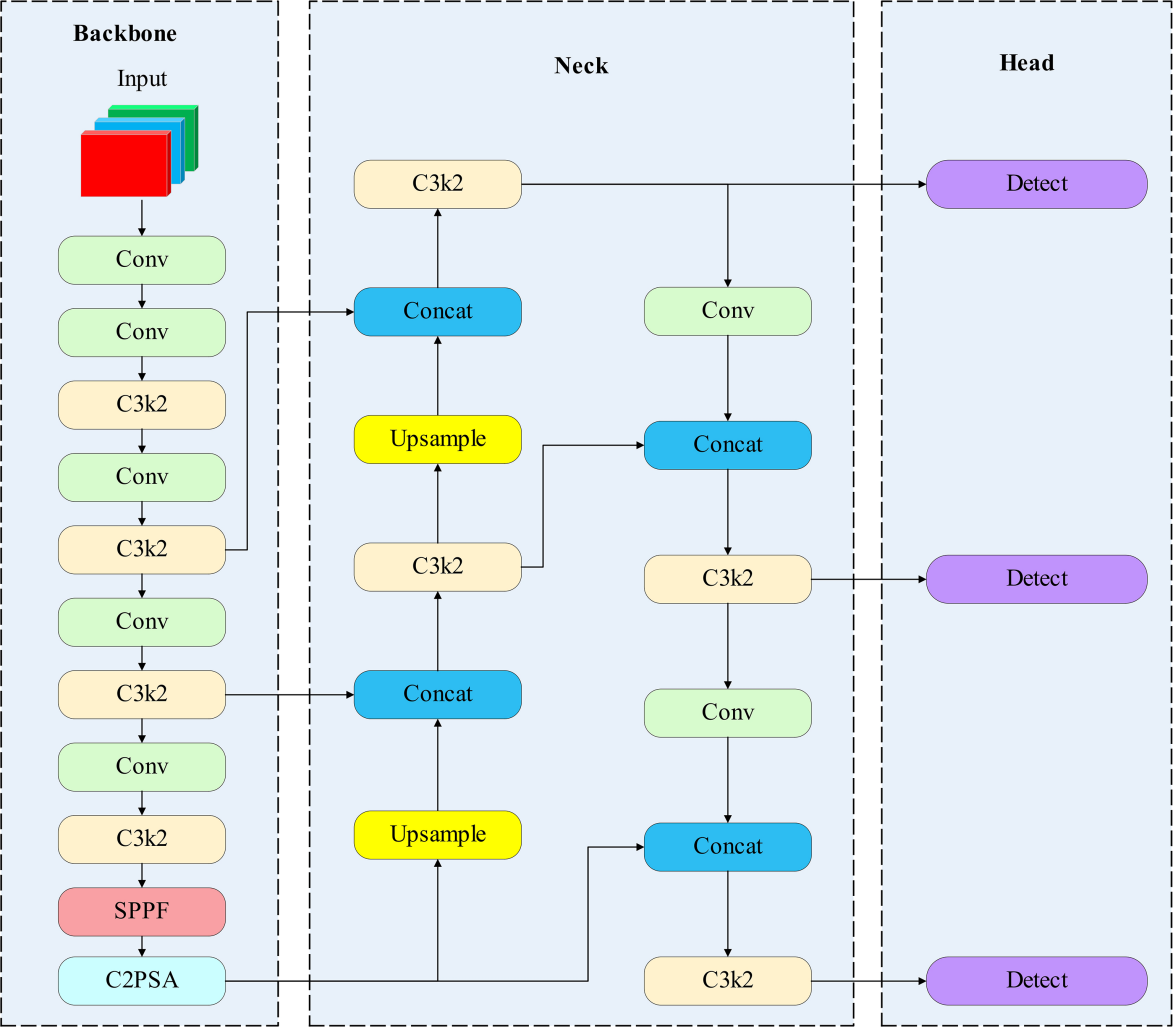
Network architecture of YOLO11n.

The architecture of YOLO11n consists mainly of three components: the backbone, neck, and detection head. The network architecture of YOLO11n is shown in **Figure 2**. The backbone is used to extract hierarchical semantic features from input maize disease images. Based on an optimized Cross Stage Partial (CSP) structure, it reduces redundant computation while maintaining stable gradient propagation through cross-stage feature splitting and fusion. At the end of the backbone, the Spatial Pyramid Pooling-Fast (SPPF) module is used to aggregate contextual information from multiple receptive fields, thereby enhancing the multi-scale representation of lesion features.

The neck is responsible for fusing multi-level features generated by the backbone. In YOLO-based detectors, bidirectional feature-fusion structures, such as PAN-FPN, are commonly used to strengthen cross-scale information interaction and improve the representation of small objects. This design is particularly important for maize disease detection, as lesions often vary substantially in size and may appear as small or inconspicuous targets in complex field backgrounds. Recent YOLO11-based studies have also shown that improved feature-fusion strategies can enhance small-object detection performance (Wang et al., 2025).

The detection head decodes the fused feature maps into category predictions and bounding-box coordinates. YOLO11n adopts a decoupled head structure, in which classification and regression are modeled by separate branches. This design reduces task interference, improves training stability, and supports more accurate lesion classification and localization while maintaining a relatively low number of parameters and low inference latency.

## 3 Corn disease detection model construction

To address the challenges in maize leaf disease detection, such as irregular lesion shapes, blurred boundaries, and low localization accuracy in complex field environments, this study proposes LSL-YOLO11n, an improved detection model based on YOLO11n. The primary improvements of this algorithm include: reconstructing the original detection head with a Lightweight Shared Convolutional Detection (LSCD) head to reduce model redundancy; introducing a Localization Quality Estimation (LQE) module to resolve regression uncertainty at blurred boundaries; and adopting the Inner-SIoU loss function to optimize bounding box regression and improve convergence and localization performance.

The novelty of the proposed LSL-YOLO11n lies in the task-oriented integration of three complementary strategies for maize leaf disease detection under complex field conditions. In contrast to many improved YOLO-based models that primarily focus on a single aspect, such as attention enhancement, feature fusion, or lightweight backbone design, the proposed framework jointly optimizes multi-scale feature representation, localization quality estimation, and bounding-box regression. Specifically, LSCD is incorporated to enhance scale-aware lesion feature extraction while reducing redundancy in the detection head. LQE is introduced to improve the consistency between classification confidence and localization quality, whereas Inner-SIoU is adopted to provide more stable regression guidance for small, irregular, and blurred lesions. These modules are designed to address different but closely related challenges in maize disease detection, thereby forming a complementary framework rather than a simple combination of existing components.

### 3.1 Lightweight shared convolutional detection head

A Lightweight Shared Convolutional Detection (LSCD) head was introduced to improve multi-scale feature representation while reducing redundancy in the detection head. The main idea of LSCD is to share convolutional parameters across feature maps of different scales. In this way, common lesion-related visual patterns, such as spots, streaks, and necrotic regions, can be captured with fewer parameters. This design is particularly suitable for maize disease detection, where lesions may appear at different scales but often share similar local textures and morphological characteristics.

**Figure 3:**
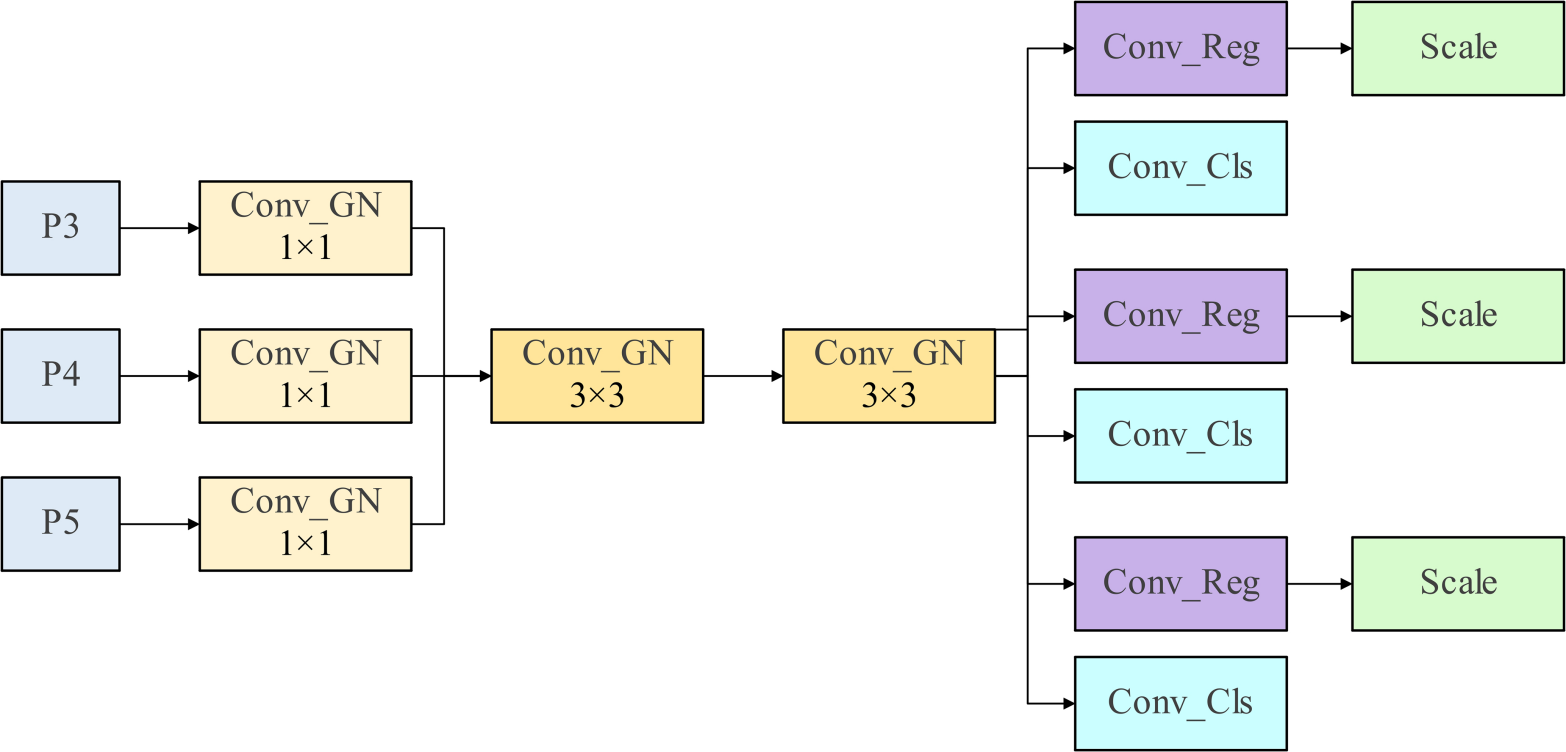
Structure of the LSCD.

As illustrated in **Figure 3**, the LSCD head receives three feature maps from the neck, denoted as P3, P4, and P5, which correspond to small-, medium-, and large-scale lesion representations, respectively. Each feature map is first processed by a scale-sensing layer to recalibrate scale-specific responses. The recalibrated features are then passed through a shared convolutional module, in which the same 3×3 convolutional kernels are applied across the three feature levels. Finally, the shared features are decoded by the classification and regression branches to predict disease categories and bounding boxes.

For the feature map at level *k*, where *k* ∈ {3, 4, 5}, the scale-sensing and shared convolution operations can be expressed as:


$${\tilde{F}}_{k}=\text{GN}\left({\text{Conv}}_{1\times 1}^{k}\left(F_{k}\right)\right),\;k\in \left\{3,4,5\right\}$$

$$H_{k}={\text{Conv}}_{3\times 3}^{\text{shared}}\left({\tilde{F}}_{k}\right),\;k\in \left\{3,4,5\right\}$$

where ${\tilde{F}}_{k}$ denotes the input feature map at scale k, ${\text{Conv}}_{1\times 1}^{k}$ represents the scale-specific recalibration operation, GN denotes Group Normalization, and ${\text{Conv}}_{3\times 3}^{\text{shared}}$ denotes the shared convolutional module used by all feature scales. Through this design, scale-specific information is preserved before weight sharing, while redundant parameters in the detection head are reduced (Wang et al., 2022b).

Overall, the LSCD head improves the efficiency of the detection head without weakening multi-scale representation. The scale-sensing layer compensates for feature distribution differences among P3, P4, and P5, whereas the shared convolutional module extracts common disease-discriminative features across scales. Therefore, the model is expected to achieve improved small-lesion detection while maintaining a lightweight structure.

### 3.2 Inner-SIoU loss function

In object detection, the design of the loss function is critical for bounding-box regression and localization accuracy. Traditional IoU-based losses, such as GIoU and CIoU, mainly consider overlap area, center distance, and aspect-ratio consistency between predicted and ground-truth boxes. However, these losses do not explicitly emphasize core-region alignment or directional guidance. As a result, the predicted boxes may follow unstable regression paths, particularly during the early training stage when they are far from the ground-truth boxes.

To address this limitation, Inner-SIoU was adopted as the bounding-box regression loss in this study. Inner-SIoU combines the auxiliary inner-box mechanism of Inner-IoU with the angle-aware geometric constraints of SIoU. Specifically, Inner-IoU generates auxiliary inner boxes by scaling the original predicted and ground-truth boxes, allowing the regression process to focus more on the core overlapping region and improving the adaptability of IoU-based losses to different regression samples (Zhang et al., 2023). In contrast, SIoU introduces an angle cost based on the direction of the center-point offset between the predicted and ground-truth boxes. This angle-aware constraint reduces unnecessary degrees of freedom in regression and guides the predicted box toward the target box along a more stable optimization path (Gevorgyan, 2022).

**Figure 4:**
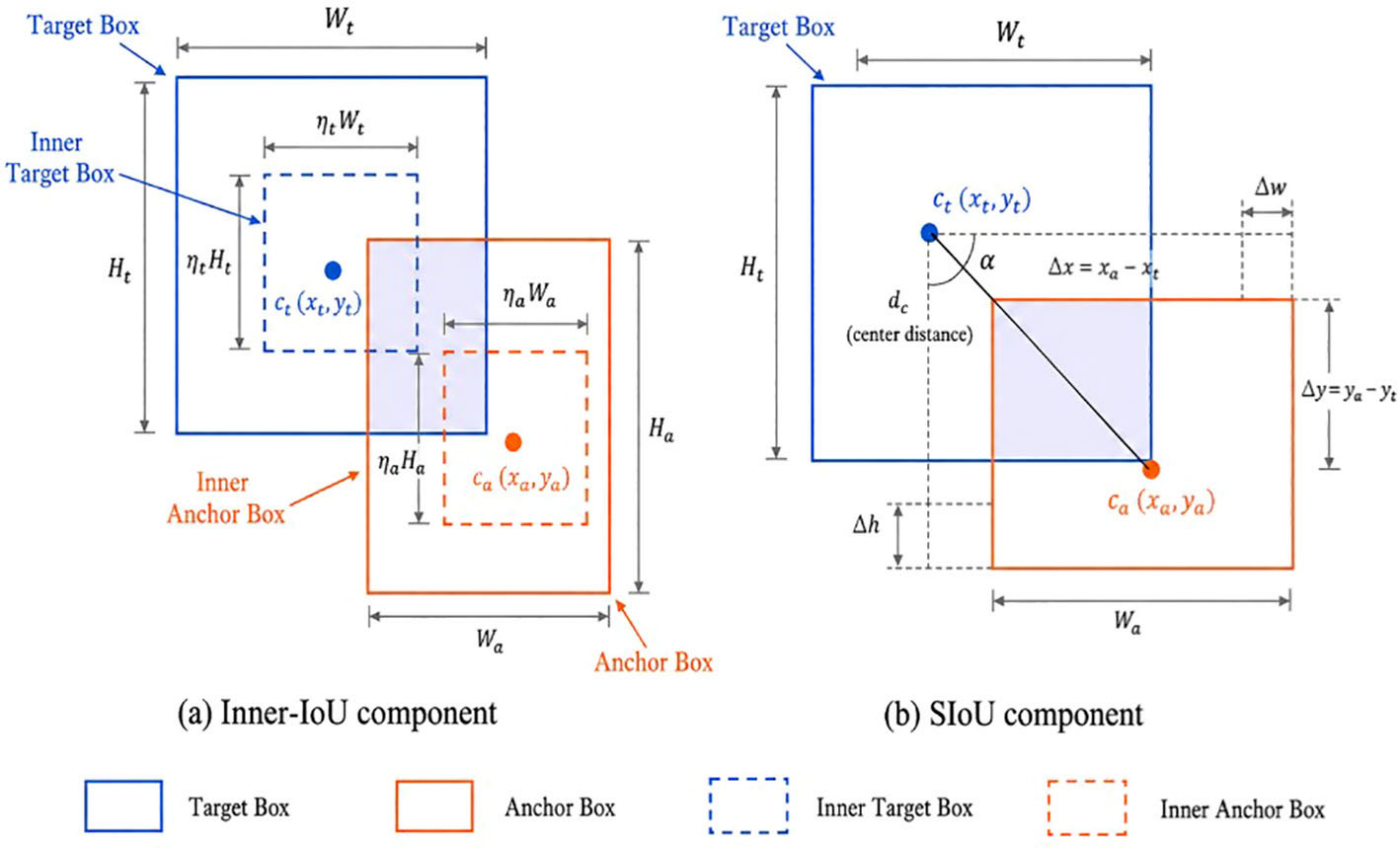
Illustration of the Inner-SIoU loss function. Panels **(a)** and **(b)** show the Inner-IoU and SIoU components, respectively.

The principle of Inner-SIoU is shown in **Figure 4**. In **Figure 4a**, the target box and anchor box are scaled to generate the inner target box and inner anchor box, respectively. These auxiliary boxes encourage the loss function to focus on the core region of the object, rather than relying only on the overlap between the original bounding boxes. In **Figure 4b**, the SIoU component models the geometric relationship between the target box and anchor box by considering center distance, horizontal and vertical offsets, angle cost, and shape-related differences. By jointly incorporating core-region overlap and direction-aware geometric constraints, Inner-SIoU provides more informative regression guidance than conventional IoU-based losses.

In maize disease detection, Inner-SIoU is expected to improve the stability and precision of bounding-box regression. Disease lesions often have irregular shapes, blurred boundaries, large scale variations, and dense spatial distributions under complex field conditions. By emphasizing core-region alignment and introducing directional constraints, Inner-SIoU can reduce unnecessary regression wandering, accelerate convergence, and improve localization accuracy for small or irregular lesions. It should be noted that Inner-SIoU and LQE serve different but complementary functions. Inner-SIoU is used during training to optimize bounding-box regression, whereas LQE is used during inference to estimate localization reliability and calibrate the final detection score.

### 3.3 Localization quality estimation based on generalized distribution

Accurate localization is essential for reliable maize disease detection. However, conventional bounding-box regression methods usually treat box coordinates as deterministic values and therefore ignore the uncertainty associated with boundary prediction. This limitation may cause boxes with high classification confidence to be retained even when their localization accuracy is poor, which can reduce overall detection reliability. To alleviate this problem, a Localization Quality Estimation (LQE) module inspired by Generalized Focal Loss V2 (GFLv2) was introduced into the YOLO11n framework (Li et al., 2021).

In the proposed framework, bounding-box regression is modeled as a discrete probability distribution rather than a single deterministic coordinate prediction. Specifically, the four boundary directions of a bounding box, namely left, right, top, and bottom, are represented by discrete probability distributions. For a specific boundary direction, the predicted boundary distance is calculated as the expectation of the corresponding distribution:


$$\hat{d}=\sum_{i=0}^{K}p_{i}d_{i}$$

Where *d_i_* denotes the *i*-th discrete candidate distance, *p_i_* represents the predicted probability assigned to *d_i_*. This formulation enables the model to represent boundary uncertainty more effectively and improves the stability of bounding-box regression, especially for lesions with blurred or irregular boundaries.

Based on the predicted boundary distributions, the LQE module further estimates the localization quality of each predicted box. Representative statistical information is extracted from the bounding-box distributions and used to generate a localization quality score. During inference, this score is combined with the classification confidence to produce the final detection score:


$$S_{final}=S_{cls}\times q_{loc}$$

Where *s_cls_* denotes the classification confidence, *q_loc_* denotes the localization quality score estimated by the LQE module, and S_final is the final confidence score used for candidate ranking and Non-Maximum Suppression (NMS). In this way, the model does not rely only on classification confidence when selecting final predictions. Predicted boxes with high classification scores but poor localization quality can be suppressed, while boxes with both reliable category prediction and accurate localization are more likely to be retained.

Intuitively, the classification branch answers “what disease category is present,” whereas the LQE module answers “how accurately the lesion is localized.” Combining these two factors improves the consistency between disease classification and bounding-box localization, especially for small lesions and blurred boundaries in field maize images.

**Figure 5:**
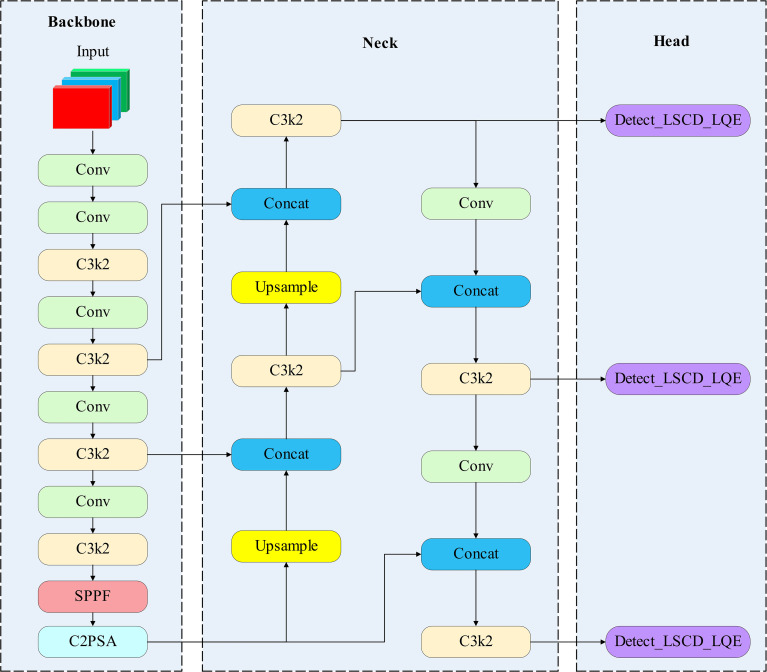
Structure of the YOLO11n_LSCD_LQE.

As illustrated in **Figure 5**, the LSCD module first generates scale-aware shared features from P3, P4, and P5. These features are then fed into two branches. The classification branch predicts disease category confidence, whereas the regression branch predicts bounding-box boundary distributions. The LQE module estimates localization quality from these distributions and combines it with the classification confidence to obtain the final detection score used during candidate ranking and NMS.

### 3.4 Experimental environment and parameter settings

The experimental environment and hyperparameter configurations used in this study are detailed in **Table 2**. The initial learning rate was set to 0.01 and adjusted using the default YOLO learning-rate decay strategy. During inference and visualization, the confidence threshold was set to 0.25, and the NMS IoU threshold was set to 0.7. The same training and inference settings were used for all comparative and ablation experiments to ensure fair comparison.

**Table 2:** Experimental environment and parameters.

Parameter	Configuration
Deep Learning Framework	PyTorch 2.1.2
Language	Python 3.10.18
Acceleration	CUDA 12.1
Optimizer	SGD
img-size	640
epochs	200
batch-size	16
Initial learning rate	0.01
Learning-rate schedule	Default YOLO learning-rate decay strategy
Confidence threshold	0.25
NMS IoU threshold	0.7

### 3.5 Evaluation metrics

To objectively evaluate the recognition performance of the model in the maize disease object detection task, Recall (R), Precision (P), mean Average Precision (mAP), and F1-score were selected as the performance evaluation metrics. These metrics enable a quantitative analysis of the model’s comprehensive performance from the perspectives of detection completeness and accuracy.

Specifically, Recall (R) represents the proportion of actual maize disease targets that are correctly detected by the model. This metric primarily reflects the model’s capability to identify disease targets; a higher R-value indicates a lower rate of missed detections. Precision (P) represents the proportion of true disease targets among all instances predicted as such by the model. This metric measures the accuracy of the detection results, with a higher P-value indicating fewer false positive errors. The mean Average Precision (mAP) comprehensively calculates the detection precision across various disease categories, providing an overall evaluation of the model’s performance in the multi-class maize disease detection task. The F1-score, serving as the harmonic mean of Precision and Recall, balances the model’s sensitivity to both missed and false detections, thus offering a comprehensive reflection of the detection performance. The specific calculation formulas are as follows:


$$\text{R}=\frac{\text{TP}}{\text{TP}+\text{FN}}\times 100\%$$

$$\text{P}=\frac{\text{TP}}{\text{TP}+\text{FP}}\times 100\%$$

$$\text{mAP}=\frac{\sum_{\text{k}=1}^{\text{N}}\text{P}(\text{k})\Delta \text{R}(\text{k})}{\text{C}}\times 100\%$$

$$\text{F}1=\frac{\text{P}\times \text{R}\times 2}{\text{P}+\text{R}}\times 100\%$$

Where TP represents the number of samples correctly identified by the model as maize diseases, FP denotes the number of samples incorrectly identified as disease targets, and FN indicates the number of disease samples not correctly detected. N is the total number of samples in the test set, P(k) represents the detection precision corresponding to the k-th sample, ΔR(k) is the change in recall between adjacent samples, and C denotes the number of maize disease categories.

## 4 Results and analysis

### 4.1 Ablation study on improved modules

To verify the effectiveness of each proposed module in the maize disease detection task, this study conducted an ablation experiment using YOLO11n as the baseline model. Under the conditions of maintaining consistent training datasets, training strategies, and hyperparameter settings, modules such as LSCD, LQE, and Inner-SIoU were sequentially introduced to systematically evaluate their individual and combined contributions to model performance. The experimental results are summarized in **Table 3**.

**Table 3:** Comparison of test results between the original and improved models.

Model	P	R	mAP
YOLO11n	0.828	0.707	0.802
YOLO11n + LSCD	0.830	0.714	0.808
YOLO11n + LQE	0.838	0.711	0.813
YOLO11n + Inner-SIoU	0.850	0.716	0.818
YOLO11n + LQE + Inner-SIoU	0.844	0.703	0.810
YOLO11n + LSCD + Inner-SIoU	0.843	0.728	0.825
YOLO11n + LSCD + LQE	0.836	0.736	0.825
YOLO11n + LSCD + LQE + Inner-SIoU	0.844	0.739	0.833

As summarized in **Table 3**, the baseline YOLO11n model achieves a Precision (P) of 0.828, Recall (R) of 0.707, and mean Average Precision (mAP) of 0.802, serving as the reference for subsequent ablation experiments. Introducing the LSCD module results in a modest improvement (P = 0.830, R = 0.714, mAP = 0.808), indicating that LSCD enhances feature representation and enables more effective extraction of local discriminative features in maize disease detection.

When the LQE module is added individually, the model attains P = 0.838, R = 0.711, and mAP = 0.813, suggesting that LQE improves feature quality and information utilization, thereby enhancing the model’s ability to distinguish between different disease types. Incorporation of the Inner-SIoU loss function alone further boosts performance, yielding P = 0.850, R = 0.716, and mAP = 0.818. This highlights the effectiveness of Inner-SIoU in refining bounding box regression through core-region emphasis and consideration of distance, shape, and directional consistency, which accelerates convergence and improves localization accuracy.

The combination of LQE and Inner-SIoU (without LSCD) achieves P = 0.844, R = 0.703, and mAP = 0.810, indicating that although both modules contribute positively, their joint effect in the absence of LSCD is somewhat limited. In contrast, integrating LSCD with Inner-SIoU produces P = 0.843, R = 0.728, and mAP = 0.825, demonstrating a strong synergistic effect between feature enhancement and localization optimization.

Finally, the full model, incorporating LSCD, LQE, and Inner-SIoU, achieves the highest overall performance with P = 0.844, R = 0.739, and mAP = 0.833. These results clearly demonstrate that the combined deployment of all three modules yields a substantial synergistic gain, optimizing both feature extraction and bounding box localization. Collectively, the ablation study validates the effectiveness of each proposed module and confirms the rationality of the integrated improvements for maize disease detection.

### 4.2 Comparison with mainstream object detection models

To further evaluate the competitiveness of the proposed LSL-YOLO11n model, comparative experiments were conducted with several mainstream object detection models, including YOLOv8, YOLOv9, YOLOv10n, and YOLOv12. All models were trained and tested on the same maize leaf disease dataset under identical experimental settings to ensure a fair comparison. The quantitative results are presented in **Table 4**.

**Table 4:** Performance comparison with mainstream object detection models.

Model	P	R	mAP
YOLOv8n	0.831	0.681	0.786
YOLOv9t	0.830	0.696	0.800
YOLOv10n	0.828	0.673	0.780
YOLOv12n	0.788	0.623	0.724
LSL-YOLO11n	0.844	0.739	0.833

As shown in **Table 4**, the proposed LSL-YOLO11n achieves the best overall detection performance among all compared models, with a Precision of 0.844, Recall of 0.739, and mAP of 0.833. Compared with YOLOv8, YOLOv9, YOLOv10n, and YOLOv12, the proposed model obtains higher mAP values by 4.7, 3.3, 5.3, and 10.9 percentage points, respectively. In terms of Recall, LSL-YOLO11n also achieves the highest value, indicating that it can detect more maize disease lesions and reduce missed detections, especially for small or inconspicuous targets in complex field backgrounds.

The superior performance of LSL-YOLO11n can be attributed to the synergistic effects of the three proposed improvements. First, the LSCD head enhances multi-scale feature representation through shared convolution and scale-aware feature recalibration, which improves the detection of small lesions with large scale variations. Second, the LQE module improves the consistency between classification confidence and localization quality, allowing the model to suppress predictions with inaccurate bounding boxes even when their classification scores are high. Third, the Inner-SIoU loss provides more stable and accurate bounding-box regression by emphasizing core-region alignment and directional guidance, which is particularly beneficial for maize lesions with irregular shapes and blurred boundaries. Therefore, compared with mainstream YOLO-based detectors, the proposed LSL-YOLO11n demonstrates stronger robustness and higher localization accuracy in maize leaf disease detection.

### 4.3 Visual detection performance

**Figure 6:**
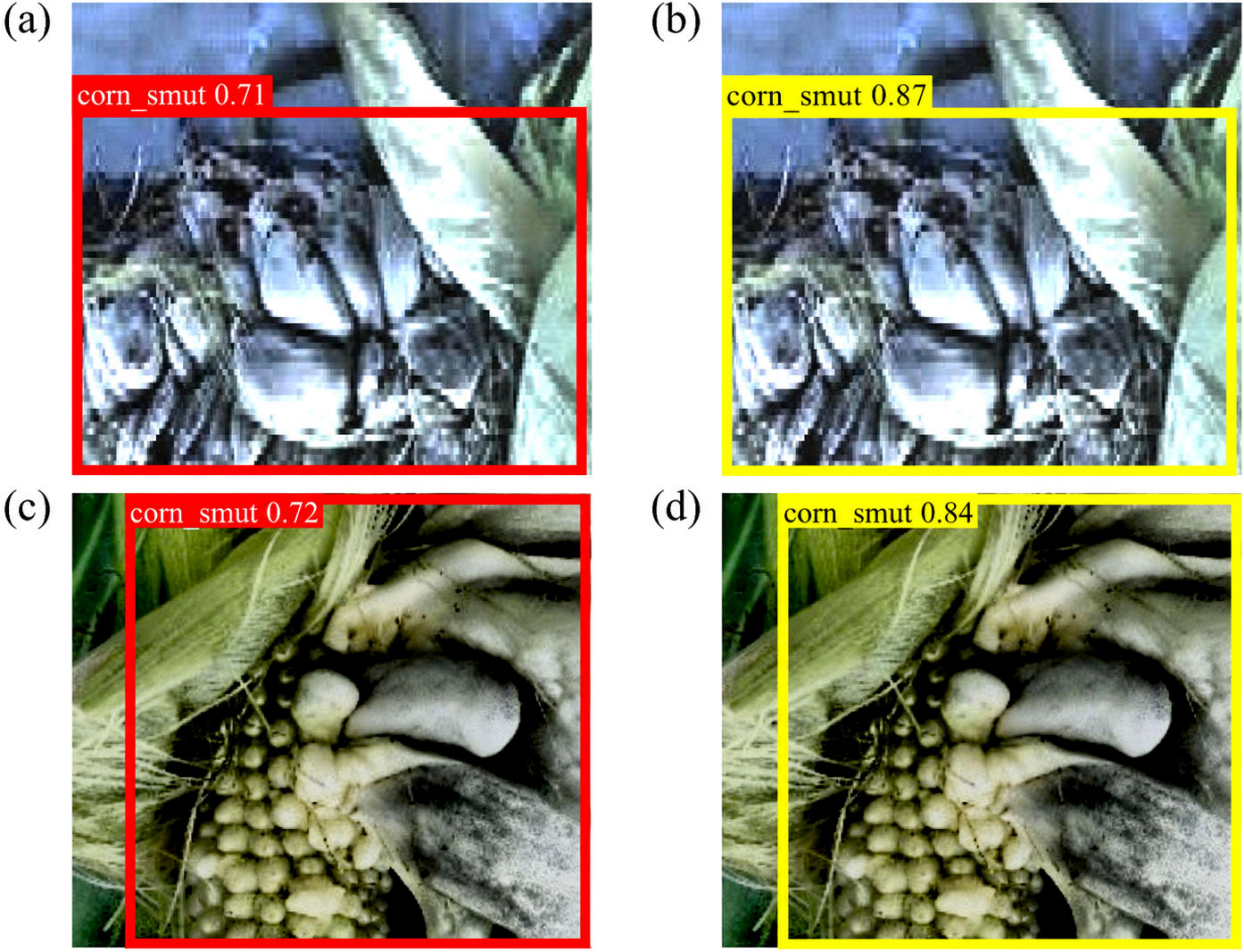
Visual comparison of maize disease detection results: **(a, c)** YOLO11n; **(b, d)** LSL-YOLO11n.

**Figure 7:**
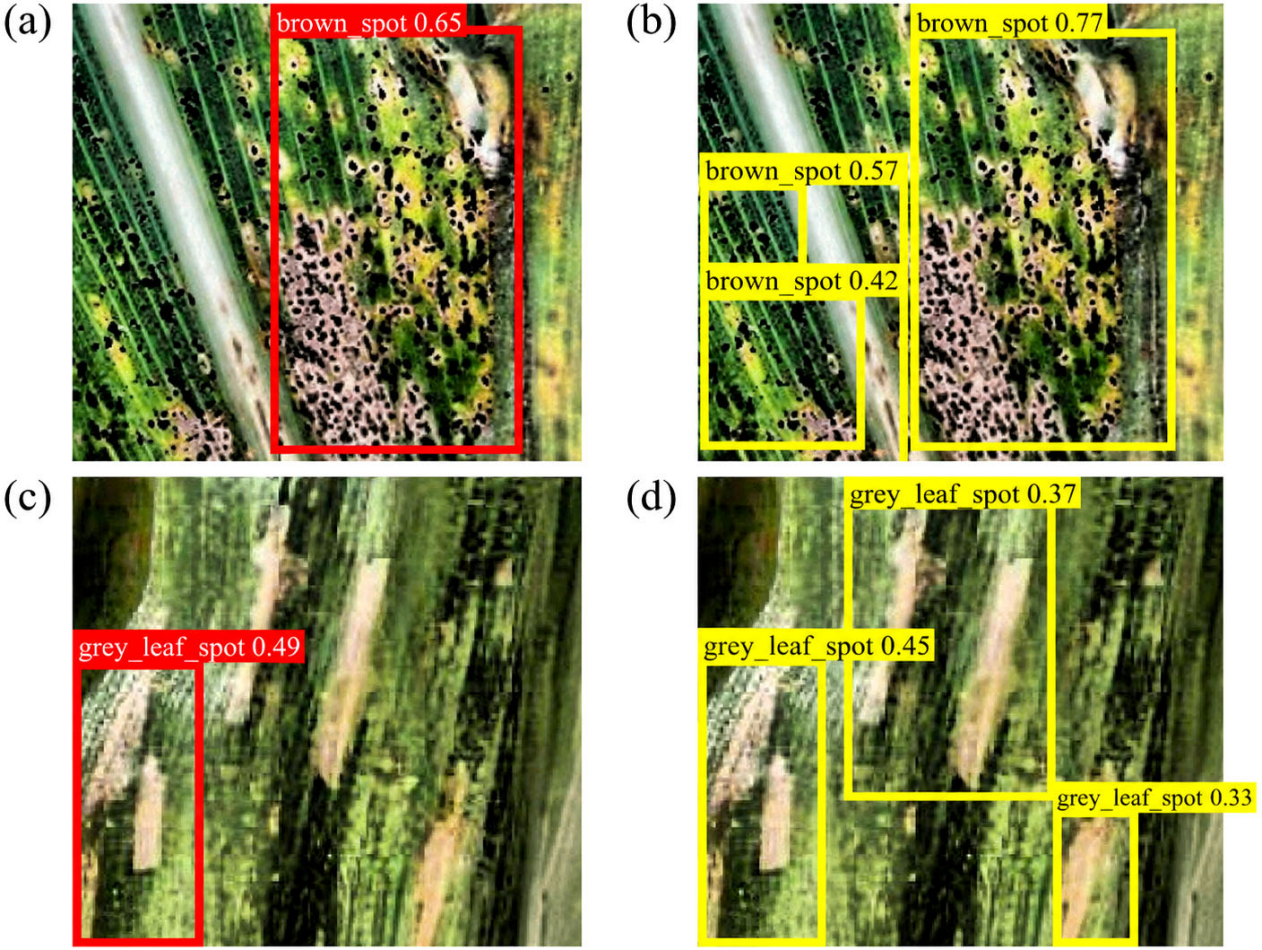
Visual comparison of maize leaf lesion detection results: **(a, c)** YOLO11n; **(b, d)** LSL-YOLO11n.

**Figure 8:**
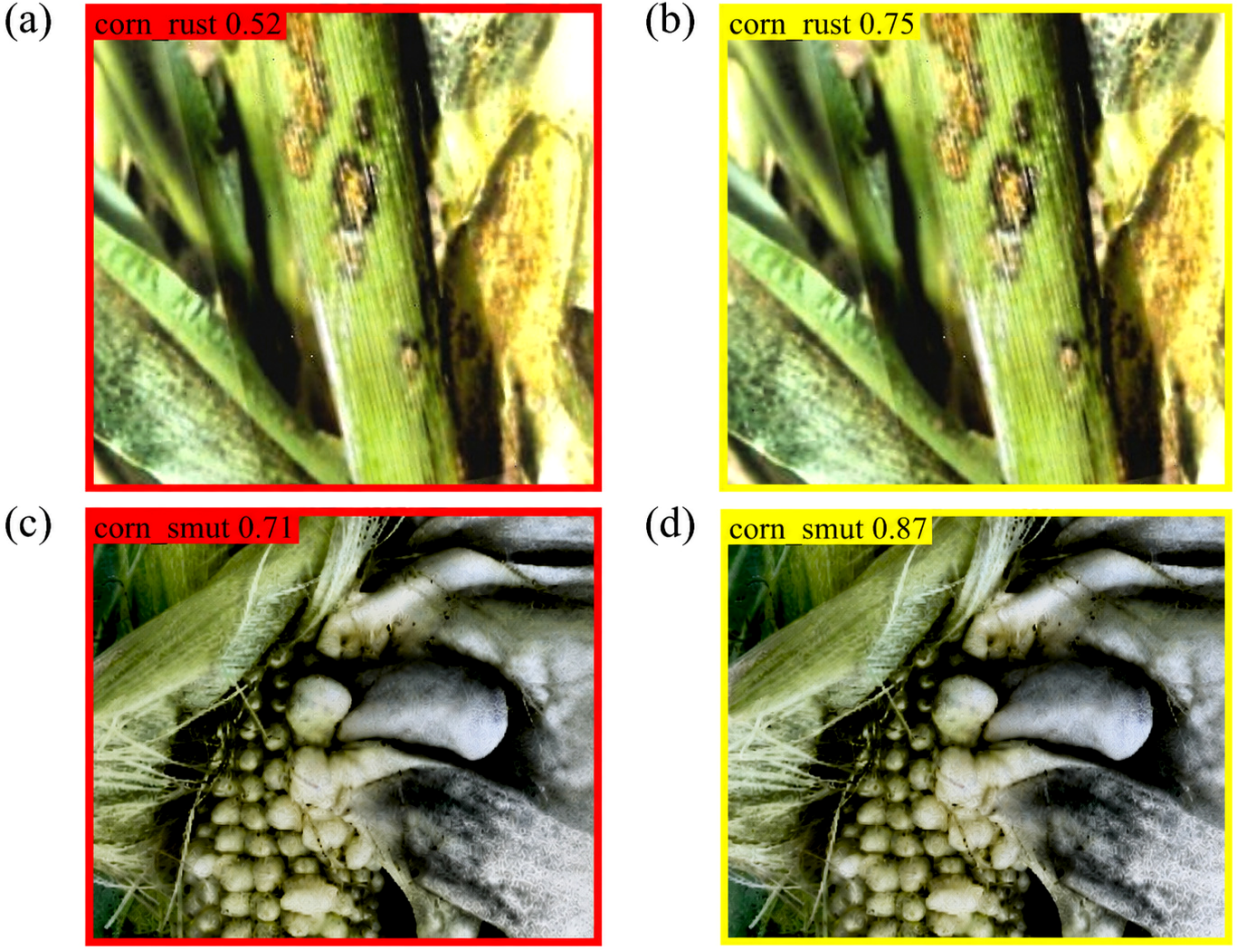
Visual comparison of detection results for different maize disease samples: **(a, c)** YOLO11n; **(b, d)** LSL-YOLO11n.

To intuitively demonstrate the actual recognition effects of the proposed LSL-YOLO11n model in the maize disease detection task, typical maize disease images from the test set were selected for visual comparative analysis. The results are shown in **Figures 6**-**8**. In each merged figure, subfigures (a,c) show the detection results of the original YOLO11n model, while subfigures (b,d) show the detection results of the improved LSL-YOLO11n model. In each figure, the left side displays the detection results of the original YOLO11n model, while the right side presents the detection results of the improved model integrating LSCD, LQE, and Inner-SIoU.

As shown in **Figure 6**, both the original YOLO11n model and the improved LSL-YOLO11n model can successfully detect corn smut lesions. However, the improved model produces higher confidence scores for the same disease category. Specifically, in **Figure 6a** and **Figure 6b**, the confidence score increases from 0.71 with the original YOLO11n model to 0.87 with the improved LSL-YOLO11n model. In **Figure 6c** and **Figure 6d**, the confidence score increases from 0.72 to 0.84. These results indicate that the improved model has stronger feature discrimination ability for corn smut lesions and can provide more confident detection results. This improvement suggests that the introduced modules enhance the representation of disease-related visual features, thereby improving the reliability and stability of lesion detection.

From the recognition results in **Figure 7**, the original YOLO11n model shows limited detection capability for small and scattered lesions in complex backgrounds. In **Figure 7a**, the original model detects only the main brown spot region with a confidence score of 0.65, whereas in **Figure 7b**, the improved LSL-YOLO11n model increases the confidence score of the main lesion to 0.77 and additionally detects several smaller lesion regions. In **Figure 7c**, the original model detects only one grey leaf spot region with a confidence score of 0.49, while in **Figure 7d**, the improved model identifies multiple lesion regions distributed in different areas of the leaf. Although some small lesions have relatively low confidence scores, their successful detection indicates that the improved model is more sensitive to small targets and scattered disease symptoms. These results suggest that the introduced LSCD module enhances multi-scale feature representation and improves the model’s ability to detect small lesions under complex visual conditions.

As shown in **Figure 8**, the improved LSL-YOLO11n model shows more stable detection performance in different maize disease samples. For the corn rust sample, the original YOLO11n model detects the lesion with a confidence score of 0.52 in **Figure 8a**, whereas the improved LSL-YOLO11n model increases the confidence score to 0.75 in **Figure 8b**. For the corn smut sample, the confidence score increases from 0.71 with the original YOLO11n model in **Figure 8c** to 0.87 with the improved model in **Figure 8d**. These results indicate that the improved model is able to provide more confident and stable detection results for different maize disease targets. This demonstrates that the introduced modules improve the robustness of the model in complex field environments.

In summary, the improved LSL-YOLO11n model demonstrates significant advantages across multiple test scenarios. Specifically, it consistently outperforms the original YOLO11n model in terms of confidence score improvement, lesion localization precision, and detection stability against complex backgrounds.

## 5 Discussion

### 5.1 Mechanism analysis of the improved modules on detection performance enhancement

By integrating LSCD, LQE, and Inner-SIoU into the YOLO11n framework, the proposed LSL-YOLO11n achieves an mAP of 0.833, which is 3.1 percentage points higher than that of the baseline model (0.802). The ablation study indicates that the LSCD module increases the mAP from 0.802 to 0.808, demonstrating the advantages of the shared convolutional detection head in multi-scale feature representation, with particularly strong performance in detecting small-scale lesions. Upon the addition of the LQE module, the mAP further increases to 0.813, yielding the most substantial gain. This indicates that LQE effectively resolves localization uncertainty caused by blurred lesion edges and irregular morphologies, thereby improving the model’s bounding box precision. The introduction of the Inner-SIoU loss further enhances detection performance. Individually, adding Inner-SIoU to YOLO11n increases the mAP to 0.818; when combined with LSCD or LQE, the mAP reaches 0.825 and 0.810, respectively. Incorporating all three modules—LSCD, LQE, and Inner-SIoU—pushes the mAP to 0.833, achieving the highest performance among all ablation experiments. By emphasizing core-region alignment and directional guidance, Inner-SIoU reduces regression path instability, accelerates convergence during early training stages, and significantly improves bounding box localization accuracy. Compared with traditional CIoU and GIoU, Inner-SIoU incorporates directional perception and core-region weighting into the loss function, enabling the model to converge more rapidly and stably. Specifically, it demonstrates marked improvements in localization precision for small lesions and in scenarios with complex backgrounds.

### 5.2 Enhancement of detection accuracy and training efficiency by inner-SIoU

The use of Inner-SIoU provides advantages in bounding box regression compared with traditional IoU-based losses. Unlike traditional loss functions such as CIoU and GIoU, Inner-SIoU mitigates the “wandering” problem of predicted boxes by emphasizing directional guidance and core-region alignment, enabling faster and more stable model convergence. The improved detection performance suggests that Inner-SIoU may provide a more effective optimization direction for bounding-box regression. In particular, when the initial predicted boxes deviate substantially from the ground truth, the directional and region-aware constraints of Inner-SIoU may help guide the predicted boxes toward more accurate localization. Furthermore, the error surface of Inner-SIoU is smoother and more stable than those of traditional loss functions, facilitating more reliable model optimization and reducing the risk of instability or overfitting during training.

During the training process, Inner-SIoU reduces the degrees of freedom in bounding box regression, allowing the model to achieve higher localization accuracy in a shorter time. It exhibits superior performance, especially in detecting small targets and handling complex backgrounds. In the current experiments, Inner-SIoU improved detection accuracy without introducing additional network structures; however, further measurements of training time and inference latency are still needed to comprehensively evaluate its computational cost. Compared to traditional methods, Inner-SIoU not only produces a higher mAP but also achieves better results within the same training duration.

### 5.3 Impact of dataset characteristics on results and generalization

The dataset employed in this study comprises eight categories (seven maize diseases and healthy leaves) and includes a total of 15,119 images with 29,366 annotated instances. The data were divided into training, validation, and test sets at a ratio of approximately 7:2:1. Despite the considerable scale, there is noticeable class imbalance. Specifically, the “streak disease” category contains only 786 annotated instances, whereas other categories, such as rust and healthy maize, are well represented. This long-tail distribution may limit the model’s ability to learn effectively from the underrepresented categories, potentially resulting in missed detections or misclassifications under varying field conditions, imaging devices, and lighting environments.

Moreover, although the dataset underwent data augmentation and manual annotation, the original images primarily originate from publicly available datasets. Consequently, discrepancies may exist between these images and actual field-acquired data in terms of background complexity, occlusion patterns, imaging distances, and angles. These differences could affect the model’s generalization to real-world scenarios. Future work should consider incorporating more real-field images collected across diverse regions and conditions to improve the robustness and cross-domain adaptability of the model.

### 5.4 Limitations and future research directions

1. Performance on real edge devices requires supplementary validation. The current experiments were primarily conducted in a desktop-level GPU environment (e.g., RTX 3090), and the deployment performance of the proposed model on real edge or embedded devices has not yet been validated. There is still a lack of evaluation regarding edge-side latency, power consumption, throughput, and practical usability on mobile or embedded devices, such as Jetson platforms, ARM CPUs, or NPUs. Therefore, future work will further verify the deployability of the model on mobile or embedded platforms when field conditions become available.
2. Insufficient coverage of complex and extreme field scenarios. Although the proposed method achieves favorable results in the maize disease detection task, there remains room for further improvement in more complex practical application scenarios. Conditions such as strong illumination, backlighting, rainy or foggy weather, overlapping leaf occlusions, and extremely small early-stage lesion targets may impose higher demands on detection stability. Future research could further expand the scale of the dataset and the types of diseases, introduce richer field-acquired data, and conduct cross-domain testing to comprehensively assess the robustness and generalization ability of the model.
3. Class imbalance and the differentiation of similar diseases. To address the issues associated with small-sample categories (e.g., streak disease) and their susceptibility to being confused with other diseases due to similarities in lesion morphology, color, and texture, strategies such as resampling, cost-sensitive learning, or semi-supervised learning can be employed. These approaches can mitigate the bias caused by the long-tail distribution and enhance the model’s discriminative ability for similar lesion categories.

## 6 Conclusion

This study proposed LSL-YOLO11n, a YOLO11n-based maize leaf disease detection model for complex field environments. The scientific novelty of this study lies in the task-oriented integration of feature representation enhancement, localization quality estimation, and bounding-box regression optimization for maize disease detection. Instead of simply stacking improved modules, the proposed model aims to address the practical challenges of small lesions, irregular lesion morphology, blurred boundaries, and complex backgrounds in field images. The results demonstrate that LSL-YOLO11n improves detection accuracy and localization stability, providing a useful technical reference for intelligent maize disease monitoring.

Experimental results on the maize disease dataset demonstrate that LSL-YOLO11n outperforms the original YOLO11n model in Precision, Recall, and mAP. The final model achieves an mAP of 83.3%, representing an improvement of 3.1 percentage points over the baseline model. The ablation results further verify the effectiveness of the improved components. These results indicate that the proposed model has practical potential for accurate maize leaf disease detection under complex field conditions.

## Data Availability

The original contributions presented in the study are included in the article/Supplementary Material. Further inquiries can be directed to the corresponding authors.
